# Supplementary material for: Differential vulnerability of the cerebellum in healthy ageing and Alzheimer’s disease
Source: Neuroimage Clin. 2021 Mar 4;30:102605. doi: 10.1016/j.nicl.2021.102605 (PMC7974323; doi:10.1016/j.nicl.2021.102605)
Supplement: Supplementary data 1 [file mmc1.docx]

**Appendix A – Details of systematic literature search**

**Table A1. Search terms for the systematic literature search.**

| **Date Searched** | **Database** | **Search Terms and Restrictions** | ***N* identified studies** |
| --- | --- | --- | --- |
| October 1st 2019 | Pubmed | (*ag*ing* OR *elderly* OR *older adults* OR *age-related*) AND (*voxel-based morphometry* OR *VBM* OR *grey matter* OR *gray matter*) in **Title/Abstract**; filter: human | 1766 |
|  | PsycInfo | (*ag*ing* OR *elderly* OR *older adults* OR *age-related*) AND (*voxel-based morphometry* OR *VBM* OR *grey matter* OR *gray matter*) in **Abstract** (filter: human, peer-reviewed journals, English) | 1078 |
| Unique records | | | 2031 |


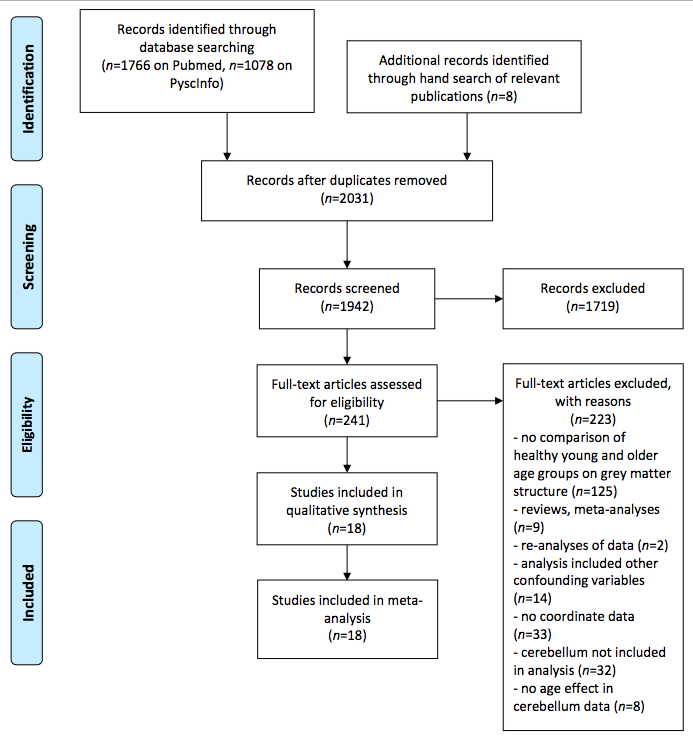


**Figure A1. PRISMA flowchart of the study selection procedure.**
