# Supplementary material for: Differential vulnerability of the cerebellum in healthy ageing and Alzheimer’s disease
Source: Neuroimage Clin. 2021 Mar 4;30:102605. doi: 10.1016/j.nicl.2021.102605 (PMC7974323; doi:10.1016/j.nicl.2021.102605)
Supplement: Supplementary data 2 [file mmc2.docx]

**Appendix B – Study Characteristics**

**Table B1. Summary of studies included in the meta-analysis of cerebellar grey matter loss in healthy ageing.**

| *Studies using direct comparisons of age groups* | | | | | | | | |  | |
| --- | --- | --- | --- | --- | --- | --- | --- | --- | --- | --- |
| **Author, year** | ***N***  **(% female)** | **Age ± *SD* (range)** | **Software** | **Preprocessing** | **Nuisance covariates; significance level** | **Foci for age effect**  **(MNI)** | **Cognitive deficits in older vs. younger adults** | | **Additional Notes** | |
| Maguire & Frith, 2003  [1] | YA  12  (50)  OA  12 (50) | YA  32±4  (23-39)  OA  75±5  (67-80) | SPM99 | Optimised  Unmodulated | *p*<.05 corrected | -47 -77 -44 | ↓ Autobiographical fact retrieval  = Memory for autobiographical events  = Memory for public events  = General knowledge | |  | |
| Steffener, Brickman, Rakitin, Gazes, & Stern, 2009  [2] | YA  37  (22)  OA  15  (53) | YA  25±4  OA  75±7 | SPM5 | Optimised  Modulated | Normalised whole brain volume  *p<*.005 uncorrected | 6 -74 -12 | ↑ Reaction times for larger set sizes in a Sternberg working memory task | |  | |
| Antonova et al., 2009  [3] | YA  10  OA  10 | YA  24±2  (20-26)  OA  72±5  (64-79) | SPM2 | Optimised  Modulated | NA  *p*<.05 FWE | -22 -58 -32  3 -75 -17  39 -69 -25  27 -57 -29  26 -48 -30^a^ | ↓ Accuracy for spatial location of objects in a spatial scene | |  | |
| Bauer, Gebhardt, Gruppe, Gallhofer, & Sammer, 2012  [4] | YA  18  OA  18 | YA  24±2  (19-28)  OA  60±6  (54-77) | SPM8 | Unified  DARTEL  Modulated | TIV  *p*<.05 FWE | 30 -66 -30  -23 -75 -23 | ↑ Reaction times during a location priming task  = Errors in location priming task | |  | |
| Kalpouzos, Persson, & Nyberg, 2012  [5] | YA  16  (50)  OA  20  (100) | YA  25  (21-39)  OA  61  (52-69) | SPM5 | Unified  Modulated | *p*<.001 FEW | -26 -35 -38 | Unclear | |  | |
| **Author, year** | ***N***  **(% female)** | **Age ± *SD* (range)** | **Software** | **Preprocessing** | **Nuisance covariates; significance level** | **Foci for age effect**  **(MNI)** | **Cognitive deficits in older vs. younger adults** | **Additional Notes** | | |
| Bauer, Sammer, & Toepper, 2018)  [6] | YA  35  (57)  OA  35  (49) | YA  27±5  (20-35)^b^  OA  61±7  (50-80)^b^ | SPM12 | Pipeline through CAT12  Modulated | TIV, gender, years of education  *p*<.05 FWE | 29 -77 -39  -32 -72 -41  -24 -45 -41  -45 -53 -45 | ↑ Errors in a high load working memory task (Corsi Block-Tapping) |  | | |
| *Studies using age as continuous variable* | | | | | | | |  | | |
| Good et al., 2001  [7] | 465  (43) | 30^c^  (17-79) | SPM99 | Optimised  Modulated | TIV, linear and nonlinear age effects  *p*<.05 corrected for multiple comparisons | 31 -90 -34 | NA |  | | |
| Alexander et al., 2006  [8] | 26  (42) | 51±16  (22-77) | SPM2 | Optimised  Modulated | TIV  *z* ≥ -2 | 38 -89 -26^a^ | No cognitive decline (MMSE>27 in all participants) | Scaled subprofile model;  only regions with negative associations with age | | |
| Abe et al., 2008  [9] | 73  (100) | 39.2±14.9  (22-70) | SPM2 | Optimised  Modulated | TIV  *p*<.05 FWE | 26 -86 -32  -24 -88 -32 | NA |  | | |
| Kalpouzos et al., 2009  [10] | 45  (53) | 49±18  (20-83) | SPM2 | Optimised  Modulated | *p*<.01 FDR  minimum cluster size *k*=20 | -46 -46 -41  36 -43 -44 | NA |  | | |
| Bergfield et al., 2010  [11] | 29^d^  (62) | 48±19  (23-84) | SPM2 | Optimised  Modulated | TIV  *z* ≥ -2 | 52 -47 -29^a^ | NA | Scaled subprofile model; only regions with negative associations with age | | |
| **Author, year** | ***N***  **(% female)** | **Age ± *SD* (range)** | **Software** | **Preprocessing** | **Nuisance covariates; significance level** | **Foci for age effect**  **(MNI)** | **Cognitive deficits in older vs. younger adults** | **Additional Notes** | | |
| Draganski et al., 2011  [12] | 26  (27) | 52  (18-85) | SPM8 | Unified,  DARTEL  Modulated | Gender, TIV  *p*<.05 FWE | -36 -58 -31  39 -58 -32 | NA |  | | |
| Salami, Eriksson, & Nyberg, 2012  [13] | 292  (52) | 60±13  (25-80) | SPM8 | Unified  DARTEL | Age and  square of orthogonalised age  TIV  *p*<.05 FDR | 35 -60 -30 | ↓ Names recalled during face-name association task | Data from Betula Prospective Study | | |
| Ziegler et al., 2012  [14] | 547  (56) | 48±17 | SPM8 | Optimised  Modulated | Scanning site, linear and quadratic age effects  *p*<.05 FWE | 15 -54 -17  20 -61 -57  -20 -55 -18  -20 -79 -35  6 -72 -44 | NA | Non-linear negative age effect | | |
| Thürling et al., 2014)  [15] | 34  (56) | 42±16  (21-74) | SPM8 | SUIT normalised  Modulated | TIV  *p<*.05 FWE | -8 -67 -23  18 -74 -29  -13 -48 -43  -30 -61 -20  -35 -70 -41  -4 -75 -20  35 -73 -39  -30 -85 -25  49 -64 -27  -30 -52 -18  32 -65 -18  -13 -66 -42  -41 -76 -39  -35 -61 -50  8 -67 -25  -41 -73 -29  36 -65 -25  -11 -73 -39  49 -55 -29 | ↓ Storage and extinction of visual threat eyeblink responses; correlated with total cerebellar volume | ROI analysis of the cerebellum; coordinates in SUIT space | | |
| **Author, year** | ***N***  **(% female)** | **Age ± *SD* (range)** | **Software** | **Preprocessing** | **Nuisance covariates; significance level** | **Foci for age effect**  **(MNI)** | **Cognitive deficits in older vs. younger adults** | **Additional Notes** | | |
| Dickie et al., 2015  [16] | 80  (50) | 43^e^  (25-64) | FSL-VBM | Optimised  Modulated | NA  *p*<.05 FDR | 20 -66 -28  -48 -60 -30 | NA | Included only results from permutation testing with 20,000 iterations | | |
| Yu, Korgaonkar, & Grieve, 2017  [17] | 438^f^  (58) | 32±19  (7-86) | SPM8 | SUIT normalised  Modulated | TIV, gender,  scanning site  *p*<.05 FWE | -37 -39-39  51 -55 -31  -3 -80 -28  -31 -82 -38  34 -83 -38  -7 -65 -44  17 -31 -21  2 -67 -14  10 -66 -41 | NA | | | ROI analysis of the cerebellum; only data pertaining to GM loss, not preservation, is included |
| Hu et al., 2018  [18] | 149  (56) | 32±12  (18-72) | SPM8 | DARTEL  Modulated | NA  *p<*.05 FWE | -35 -82 -39  -8 -70 -14  -39 -40 -42  38 -40 -42  38 -79 -39 | ↑ Reaction time in a stop signal task | | |  |

^a^Coordinates transformed from Talairach & Tournaux to MNI

^b^Numbers based on mean of high and low performing cognitive group

^c^Mean of four medians from four groups of participants (right-handed females, right-handed males, left-handed females, left-handed males)

^d^Only data from Group 1 included because Group 2 was assessed in Alexander et al. (2006)

^e^Median age

^f^Total study sample size minus 41 subjects who were excluded from the VBM analysis

Abbreviations. CAT: computational anatomy toolbox. DARTEL: diffeomorphic anatomical registration trough exponentiated lie algebra; FDR: false discovery rate. FWE: familywise error; FSL: functional magnetic imaging of the brain (FMRIB) software library; GM: grey matter. MA: middle-aged adults. MNI: Montreal Neurological Institute; N: sample size; NA: not applicable; OA: older adults. ROI: region of interest. SD: Standard Deviation. SPM: Statistical Parametric Mapping. SUIT: spatially unbiased infratentorial template. TIV: total intracranial volume. VBM: voxel-based morphometry. YA: younger adults.

**Table B2. Study characteristics of records included in the coordinate-based meta-analysis.** Studies are listed according to the degree of cognitive impairment going from least to most severe as measured using the MMSE. Note that this table is based on Gellersen et al. (2017)*: <https://jnnp.bmj.com/content/88/9/780.full#DC1>.

| **Authors** | **Notes on diagnosis** | ***N* patients (% female)** | ***N* controls (% female)** | **Age patients**  **± *SD*** | **Age controls**  **± *SD*** | ***p*-value age difference** | **MMSE or ACE patients**  **± *SD*** | **Disease Duration**  **(years ± *SD*)** | **Coordinates (MNI)** | **Relationship between cerebellar grey matter and cognition and clinical ratings**  **(NA if no such analysis was carried out)** | **Cognitive deficits in patients vs. controls** |
| --- | --- | --- | --- | --- | --- | --- | --- | --- | --- | --- | --- |
| Farrow et al., 2007  [19] | Probable AD based on NINCDS-ADRDA criteria | 7 | 11 | 78±7 | 71±4 | .014 | 25±4 (MMSE) | 4 | 25 -40 -29  -24 -36 -29 | No correlation between ADAS-TES/MMSE and cerebellar GM | ↓ MMSE, ADAS-TES |
| Mazère et al., 2008  [20] | Probable based on NINCDS-ADRDA criteria | 8 (63) | 8 (75) | 80±7 | 74±3 | NS | 24±2 (MMSE) | NA | -44 -65 -42  27 -66 -11 | NA | ↓ MMSE |
| Ossenkoppele et al., 2015  [21] | Subgroup of AD patients defined as typical AD based on NIA-AA criteria; biomarker confirmed | 58 (39) | 61 (38) | 64±9 | 64±8 | NS | 23±4 (MMSE) | NA | -39 -82 -33  46 -73 -36 | NA | ↓ MMSE  47% of patients with memory impairment  7% with executive functioning |
| Canu et al., 2011  [22] | Based on NINCDS-ADRDA criteria | 17 (82) | 13 (46) | 77±6 | 73±7 | NS | 21±5 (MMSE) | NA | 42 -59 -25  32 -64 -36  -29 -70 -39  -36 -67 -32 | NA | ↓ MMSE |
| Möller et al., 2013  [23] | Subgroup of late-onset probable AD based on NINCDS-ADRDA criteria | 120 (46) | 71 (50) | 72±5 | 71±4 | NS | 21±5 (MMSE) | NA | 33 -60 -27  30 -69 -38  12 -61 -23  26 -49 -47  -26 -48 -45  -34 -48 -45  -30 -42 -42  10 -67 -36 | No correlation between MMSE and cerebellar GM | ↓ MMSE  ↓ RAVLT immediate and delayed  ↓ Trail Making Test A and B |
| Colloby, O’Brien, & Taylor, 2014  [24] | Probable AD based on NINCDS-ADRDA criteria | 47 | 39 | 79±9 | 77±6 | NS | 21±4 (MMSE) | NA | -33 -43 -24  42 -43 -26 | No correlation between MMSE and cerebellar GM | ↓ MMSE  ↓ CAMCOG |
|  |  |  |  |  |  |  |  |  |  |  |  |
| **Authors** | **Notes on diagnosis** | ***N* patients (% female)** | ***N* controls (% female)** | **Age patients**  **± *SD*** | **Age controls**  **± *SD*** | ***p*-value age difference** | **MMSE or ACE patients**  **± *SD*** | **Disease Duration**  **(years ± *SD*)** | **Coordinates (MNI)** | **Relationship between cerebellar grey matter and cognition and clinical ratings**  **(NA if no such analysis was carried out)** | **Cognitive deficits in patients vs. controls** |
| Canu et al., 2012  [25] | Subgroup of late-onset probable AD based on NINCDS-ADRDA criteria | 24 (67) | 24 (71) | 78±5 | 76±4 | NS | 21±4 (MMSE) | 4±2 | 33 -75 -28 | NA | ↓ MMSE  ↓ RCFT delayed  ↓ RAVLT immediate and delayed  ↓ Trail Making Test |
| Toniolo et al., 2018 [26] | Probable AD based on NINCDS-ADRDA criteria | 53 (66) | 34 (50) | 75±6 | 69±7 | NS | 20±3 (MMSE) | NA | 19 -35 -19  39 -63 -23  -4 -51 -26 | Correlation between anterior and posterior cerebellar grey matter volume and Copy of drawings test; no correlation with MMSE, short-term memory, long-term memory, word fluency, language or executive functions. | ↓ MMSE  ↓ RAVLT immediate and delayed  ↓ Phonological verbal fluency  ↓ Digit span forward and backward  ↓ Copy of drawings and drawings with landmarks  ↓ Raven’s progressive matrices  ↓ Corsi blocking task |
| Lehmann et al., 2011  [27] | Probable AD based on NINCDS-ADRDA criteria; typical AD presentation. | 30 (53) | 50 (66) | 69±9 | 63±10 | <.005 | 19±5 (MMSE) | 5 | 8 -49 -30 | NA | ↓ MMSE |
| Serra et al., 2014  [28] | Probable AD based on NINCDS-ADRDA criteria | 48 (35) | 20 (65) | 71±6 | 70±6 | NS | 19±3 (MMSE) | 4±3 | -12 -86 -24 | NA | ↓ MMSE  ↓ RAVLT immediate and delayed  ↓ RCFT delayed  ↓ Short story test  ↓ Corsi blocking task  ↓ Phonological word fluency  ↓ Card sorting test  ↓ Raven’s progressive matrices |
| **Authors** | **Notes on diagnosis** | ***N* patients (% female)** | ***N* controls (% female)** | **Age patients**  **± *SD*** | **Age controls**  **± *SD*** | ***p*-value age difference** | **MMSE or ACE patients**  **± *SD*** | **Disease Duration**  **(years ± *SD*)** | **Coordinates (MNI)** | **Relationship between cerebellar grey matter and cognition and clinical ratings**  **(NA if no such analysis was carried out)** | **Cognitive deficits in patients vs. controls** |
| Guo et al., 2016  [29] | Probable AD based on NINCDS-ADRDA criteria | 34 (44) | 34 (53) | 62±6 | 64±5 | NS | NA (MMSE) | 3±3 | -32 -72 -29  -31 -60 -19  27 -71 -28  27 -76 -26 | NA | ↓ RAVLT  ↓ RCFT  ↓ Doors and people test |
| Raji et al., 2009  [30] | Probable AD based on NINCDS-ADRDA criteria | 33 (33) | 169 (57) | 83±5 | 78±4 | .001 | 76±13^a^ (MMSE) | NA | -24 -33 -31  28 -33 -34  1 -37 -20 | NA | ↓ MMSE |
| Ahmed et al., 2019 [31] | Probable AD based on NINCDS-ADRDA criteria | 16 (38) | 19 (32) | 60±6 | 63±7 | NS | 62±16 (ACE-III) | 4±2 | -52 -58 -46  42 -52 -58 | NA | ↓ ACE-III |

^a^Use of modified MMSE.

Abbreviations. ACE-III: Addenbrookes Cognitive Examination – Version 3. AD: Alzheimer’s disease. ADAS-TES: Alzheimer's Disease Assessment Scale Total Error Score. CAMCOG: Cambridge Cognitive Examination. MMSE: Mini Mental State Exam. MNI: Montreal Neurological Institute. NA: not available. NIA-AA: National Institute on Aging-Alzheimer’s Association. NINCDS-ADRDA: National Institute of Neurological and Communicative Disorders and Stroke and Alzheimer's Disease and Related Disorders Association. NS: not significant. RAVLT: Rey Auditory Verbal Learning Test. RCFT: Rey Complex Figure Test. SD: Standard Deviation.

**References**

[1] Maguire EA, Frith CD. Aging affects the engagement of the hippocampus during autobiographical memory retrieval. Brain 2003;126:1511–23. doi:10.1093/brain/awg157.

[2] Steffener J, Brickman AM, Rakitin BC, Gazes Y, Stern Y. The impact of age-related changes on working memory functional activity. Brain Imaging Behav 2009;3:142–53. doi:10.1007/s11682-008-9056-x.

[3] Antonova E, Parslow D, Brammer M, Dawson GR, Jackson SHDD, Morris RG. Age-related neural activity during allocentric spatial memory. Memory 2009;17:125–43. doi:10.1080/09658210802077348.

[4] Bauer E, Gebhardt H, Gruppe H, Gallhofer B, Sammer G. Altered negative priming in older subjects: First evidence from behavioral and neural level. Front Hum Neurosci 2012;6:1–10. doi:10.3389/fnhum.2012.00270.

[5] Kalpouzos G, Persson J, Nyberg L. Local brain atrophy accounts for functional activity differences in normal aging. Neurobiol Aging 2012;33:e1–13. doi:10.1016/j.neurobiolaging.2011.02.021.

[6] Bauer E, Sammer G, Toepper M. Performance level and cortical atrophy modulate the neural response to increasing working memory load in younger and older adults. Front Aging Neurosci 2018;10:1–15. doi:10.3389/fnagi.2018.00265.

[7] Good CD, Johnsrude IS, Ashburner J, Henson RNA, Friston KJ, Frackowiak RSJ. A voxel-based morphometric study of ageing in 465 normal adult human brains. Neuroimage 2001;14:21–36. doi:10.1006/nimg.2001.0786.

[8] Alexander GE, Chen K, Merkley TL, Reiman EM, Caselli RJ, Aschenbrenner M, et al. Regional network of magnetic resonance imaging gray matter volume in healthy aging. Neuroreport 2006;17:951–6. doi:10.1097/01.wnr.0000220135.16844.b6.

[9] Abe O, Yamasue H, Aoki S, Suga M, Yamada H, Kasai K, et al. Aging in the CNS: comparison of gray/white matter volume and diffusion tensor data. Neurobiol Aging 2008;29:102–16. doi:10.1016/j.neurobiolaging.2006.09.003.

[10] Kalpouzos G, Chételat G, Baron JC, Landeau B, Mevel K, Godeau C, et al. Voxel-based mapping of brain gray matter volume and glucose metabolism profiles in normal aging. Neurobiol Aging 2009;30:112–24. doi:10.1016/j.neurobiolaging.2007.05.019.

[11] Bergfield KL, Hanson KD, Chen K, Teipel SJ, Hampel H, Rapoport SI, et al. Age-related networks of regional covariance in MRI gray matter: Reproducible multivariate patterns in healthy aging. Neuroimage 2010;49:1750–9. doi:10.1016/j.neuroimage.2009.09.051.

[12] Draganski B, Ashburner J, Hutton C, Kherif F, Frackowiak RSJJ, Helms G, et al. Regional specificity of MRI contrast parameter changes in normal ageing revealed by voxel-based quantification (VBQ). Neuroimage 2011;55:1423–34. doi:10.1016/j.neuroimage.2011.01.052.

[13] Salami A, Eriksson J, Nyberg L. Opposing effects of aging on large-scale Brain systems for memory encoding and cognitive control. J Neurosci 2012;32:10749–57. doi:10.1523/JNEUROSCI.0278-12.2012.

[14] Ziegler G, Dahnke R, Jäncke L, Yotter RA, May A, Gaser C. Brain structural trajectories over the adult lifespan. Hum Brain Mapp 2012;33:2377–89. doi:10.1002/hbm.21374.

[15] Thürling M, Galuba J, Thieme A, Burciu RG, Goricke S, Beck A, et al. Age effects in storage and extinction of a naturally acquired conditioned eyeblink response. Neurobiol Learn Mem 2014;109:104–12. doi:10.1016/j.nlm.2013.12.007.

[16] Dickie DA, Mikhael S, Job DE, Wardlaw JM, Laidlaw DH, Bastin ME. Permutation and parametric tests for effect sizes in voxel-based morphometry of gray matter volume in brain structural MRI. Magn Reson Imaging 2015;33:1299–305. doi:10.1016/j.mri.2015.07.014.

[17] Yu T, Korgaonkar MS, Grieve SM. Gray matter atrophy in the cerebellum—Evidence of increased vulnerability of the crus and vermis with advancing age. The Cerebellum 2017;16:388–97. doi:10.1007/s12311-016-0813-x.

[18] Hu S, Ide JS, Chao HH, Castagna B, Fischer KA, Zhang S, et al. Structural and functional cerebral bases of diminished inhibitory control during healthy aging. Hum Brain Mapp 2018;39:5085–5096. doi:10.1002/hbm.24347.

[19] Farrow TFD, Thiyagesh SN, Wilkinson ID, Parks RW, Ingram L, Woodruff PWR. Fronto-temporal-lobe atrophy in early-stage Alzheimer’s disease identified using an improved detection methodology. Psychiatry Res 2007;155:11–9. doi:10.1016/j.pscychresns.2006.12.013.

[20] Mazère J, Prunier C, Barret O, Guyot M, Hommet C, Guilloteau D, et al. In vivo SPECT imaging of vesicular acetylcholine transporter using [123I]-IBVM in early Alzheimer’s disease. Neuroimage 2008;40:280–8. doi:10.1016/j.neuroimage.2007.11.028.

[21] Ossenkoppele R, Pijnenburg YAL, Perry DC, Cohn-Sheehy BI, Scheltens NME, Vogel JW, et al. The behavioural/dysexecutive variant of Alzheimer’s disease: Clinical, neuroimaging and pathological features. Brain 2015;138:2732–49. doi:10.1093/brain/awv191.

[22] Canu E, McLaren DG, Fitzgerald ME, Bendlin BB, Zoccatelli G, Alessandrini F, et al. Mapping the structural brain changes in Alzheimer’s disease: The independent contribution of two imaging modalities. Adv Alzheimer’s Dis 2011;2:487–98. doi:10.3233/978-1-60750-793-2-487.

[23] Möller C, Vrenken H, Jiskoot L, Versteeg A, Barkhof F, Scheltens P, et al. Different patterns of gray matter atrophy in early- and late-onset Alzheimer’s disease. Neurobiol Aging 2013;34:2014–22. doi:http://dx.doi.org/10.1016/j.neurobiolaging.2013.02.013.

[24] Colloby SJ, O’Brien JT, Taylor JP. Patterns of cerebellar volume loss in dementia with Lewy bodies and Alzheimer’s disease: A VBM-DARTEL study. Psychiatry Res - Neuroimaging 2014;223:187–91. doi:10.1016/j.pscychresns.2014.06.006.

[25] Canu E, Frisoni GB, Agosta F, Pievani M, Bonetti M, Filippi M. Early and late onset Alzheimer’s disease patients have distinct patterns of white matter damage. Neurobiol Aging 2012;33:1023–33. doi:10.1016/j.neurobiolaging.2010.09.021.

[26] Toniolo S, Serra L, Olivito G, Marra C, Bozzali M, Cercignani M. Patterns of cerebellar gray matter atrophy across Alzheimer’s disease progression. Front Cell Neurosci 2018;12:1–8. doi:10.3389/fncel.2018.00430.

[27] Lehmann M, Crutch SJ, Ridgway GR, Ridha BH, Barnes J, Warrington EK, et al. Cortical thickness and voxel-based morphometry in posterior cortical atrophy and typical Alzheimer’s disease. Neurobiol Aging 2011;32:1466–76. doi:10.1016/j.neurobiolaging.2009.08.017.

[28] Serra L, Fadda L, Perri R, Spanò B, Marra C, Castelli D, et al. Constructional apraxia as a distinctive cognitive and structural brain feature of pre-senile Alzheimer’s disease. J Alzheimer’s Dis 2014;38:391–402. doi:10.3233/JAD-130656.

[29] Guo CC, Tan R, Hodges JR, Hu X, Sami S, Hornberger M. Network-selective vulnerability of the human cerebellum to Alzheimer’s disease and frontotemporal dementia. Brain 2016;139:1527–38. doi:10.1093/brain/aww003.

[30] Raji CA, Lopez OL, Kuller LH, Carmichael OT, Becker JT. Age, Alzheimer disease, and brain structure. Neurology 2009;73:1899–905. doi:10.1212/WNL.0b013e3181c3f293.

[31] Ahmed RM, Landin-Romero R, Liang CT, Keogh JM, Henning E, Strikwerda-Brown C, et al. Neural networks associated with body composition in frontotemporal dementia. Ann Clin Transl Neurol 2019;6:1707–17. doi:10.1002/acn3.50869.
